# Effect of overweight and obesity on serum interleukin-6 in children and adolescents with bronchial asthma

**DOI:** 10.7555/JBR.38.20240381

**Published:** 2025-07-12

**Authors:** Regina Niyazovna Khramova, Tatyana Ivanovna Eliseeva, Dmitriy Yuryevich Ovsyannikov, Maksim Aleksandrovich Karpenko, Elena Valerjevna Tush, Svetlana Viktorovna Krasilnikova, Daniil Vitalievich Azarnov, Sofya Alekseevna Teremova, Nailya Iskhakovna Kubysheva, Оlga Vladimirovna Khaletskaya, Natalia Anatolievna Geppe

**Affiliations:** 1 Department of Hospital Pediatrics, Privolzhsky Research Medical University of the Ministry of Health of the Russian Federation, Nizhny Novgorod 603005, Russian Federation; 2 Department of Pediatrics, Medical Institute, Peoples' Friendship University of Russia (RUDN University), Moscow 117198, Russian Federation; 3 Neurocognitive Research Laboratory, Kazan Federal University, Kazan 420000, Russian Federation; 4 Clinical Institute of Children’s Health named after N.F. Filatov, Sechenov First Moscow State Medical University, Moscow 119992, Russian Federation

Dear Editor,

Bronchial asthma (BA) is a common chronic inflammatory disease of the airways, characterized by variable obstruction and airway hyperreactivity. It is a heterogeneous lung disease characterized by variability in both clinical symptoms and the underlying cellular and molecular mechanisms^[1]^. Type 2 inflammation (T2 inflammation) is the primary pathogenic mechanism of asthma in children and adolescents; however, it may not dominate in all asthma patients. The heterogeneity of the clinical manifestations of BA and its underlying mechanisms has led to the identification of various phenotypes and endotypes of the disease^[1]^. Currently, significant attention is focused on the asthma phenotype associated with overweight and obesity^[2]^.

It is believed that a key factor contributing to the chronic course of asthma associated with obesity is low-grade systemic inflammation generated by excess adipose tissue^[3–4]^. Adipocytes and macrophages in adipose tissue secrete various pro-inflammatory cytokines, including interleukin 6 (IL-6)^[5–6]^. Increased levels of IL-6 in the serum of patients with both asthma and obesity have been reported^[7]^. However, few studies have investigated serum IL-6 concentration in children and adolescents with asthma, and none have examined the relationship between serum IL-6 levels and respiratory parameters. The aim of the current letter is to study the effect of overweight and obesity on interleukin-6 levels in children and adolescents with asthma.

The present letter reports findings from a single-center, observational, cross-sectional pilot study. Data were obtained during examinations of children and adolescents receiving treatment for atopic asthma at the State Budgetary Healthcare Institution of the Nizhny Novgorod Region "Children's City Clinical Hospital No. 1 of Nizhny Novgorod" between 2017 and 2023. Inclusion criteria were: (1) a diagnosis of asthma established in accordance with the current international consensus documents (GINA, 2016–2023)^[1]^; and (2) patient age between eight and 17 years. Exclusion criteria included: (1) body mass index (BMI) greater than +2.5 Z-score; (2) acute infectious diseases or fever; (3) diabetes mellitus, autoimmune disorders, primary immunodeficiencies, cancer, atopic dermatitis, and parasitic diseases; (4) severe asthma^[1]^; and (5) systemic use of glucocorticoids, antiepileptic drugs, nonsteroidal anti-inflammatory drugs, or angiotensin-converting enzyme inhibitors. All patients had their basic anthropometric parameters measured. Anthropometric parameters (height, body weight, and BMI) were assessed using WHO tables, taking into account the patients' sex and age. BMI was calculated as weight in kilograms divided by the square of height in meters (kg/m^2^). Based on BMI data, patients were divided into two groups: group 1 (*n* = 61), patients with underweight and normal body weight (BMI values ranging from −2 to +1 Z-score); group 2 (*n* = 42), overweight and obese patients (BMI values above +1 Z-score but less than +2.5 Z-score). Body fat percentage (BF%) was measured using a body composition monitor (Omron BF214, OMRON HEALTHCARE Co. Ltd., Kyoto, Japan). Spirometry was performed using a Masterscreen pneumospirometer (Jaeger, Germany) to assess FVC (L), the forced vital capacity of the lungs that reflects lung volume, FEV_1_ (L), the forced volume in one second, and the FEV_1_/FVC ratio, the main spirometry parameter for diagnosing obstructive disorders. zFVC, zFEV_1_, and zFEV_1_/FVC were calculated using the Global Lung Function Initiative calculator, supported by the European Respiratory Society (https://www.ersnet.org/science-and-research/ongoing-clinical-research-collaborations/the-global-lung-function-initiative/gli-tools). Obstructive respiratory disorders were diagnosed when FEV_1_/FVC Z-score values were < –1.645. The serum level of IL-6 was measured using the IgE-ELISA-Best and Interleukin-6-IFA-Best test systems produced by Vector-Best (Novosibirsk, Russia) on an automated enzyme immunoassay analyzer Radim Alisei Q.S. (RADIM Deutschland GmbH, Freiburg, Germany). The detection sensitivity for serum IL-6 was 0.1 pg/mL, with a range from 0 to 90 pg/mL. The study was carried out in accordance with the Declaration of Helsinki (2013) and approved by the Institutional Review Board of Privolzhsky Research Medical University (Protocol No. 13 dated 10.10.2016). As this was a pilot study, no sample size calculation was conducted. Data analysis was performed using Statgraphics Centurion v.16 (Statgraphics Technologies, Inc., The Plains, Virginia, USA). Quantitative indicators were evaluated for normal distribution, and data are presented as median (Q1, Q3), where Q1 and Q3 represent the first and third quartiles, respectively, because of non-normal distribution. The Mann–Whitney test was performed to compare quantitative variables in two independent groups. Correlation analysis was performed using the Spearman correlation coefficient. Differences were considered statistically significant at *P* < 0.05.

A total of 103 patients with asthma aged six to 17 years (median = 13.0, Q1 = 10.5, Q3 = 15.0 years) were examined, of whom 76% were boys (78/103). A positive correlation was found between IL-6 levels and zBMI in the overall group (*R* = 0.32, *P* = 0.001) and in the group of boys (*R* = 0.34, *P* = 0.003). Further analysis revealed a positive correlation between IL-6 and BF% in both the overall group (*R* = 0.34, *P* = 0.021) and the group of boys (*R* = 0.38, *P* = 0.028).

In underweight and normal weight patients, no statistically significant differences in serum IL-6 levels were observed between those with and without obstructive disorders (*P* = 0.250; ***Table 1***). Conversely, in the overweight and obese group, IL-6 levels were significantly higher in patients with obstructive disorders compared with those without (*P* = 0.026). In patients without bronchial obstruction, IL-6 levels were comparable in patients with different body weights. However, in patients with obstructive disorders, IL-6 levels were significantly increased in the overweight and obese group (*P* = 0.024).

**Table 1:** Serum IL-6 levels (pg/mL) in asthmatic patients categorized by body weight and presence of obstructive disorders

Groups	All (*n*=103)	Without obstructive disorders (*n*=60)	With obstructive disorders (*n*=43)	*P*-values
All patients (*n*=103)	0.69 (0.00, 1.94)	0.58 (0.00, 1.34)	1.35 (0.36, 3.27)	0.011
Patients with underweight and normal body weight (*n*=61)	0.59 (0.05, 1.51)	0.54 (0.00, 1.26)	0.63 (0.28, 1.92)	0.250
Overweight and obese patients (*n*=42)	1.15 (0.00, 3.27)	0.77 (0.00, 1.65)	1.74 (0.68, 5.41)	0.026
*P*-values	0.023	0.650	0.024	–
Data are presented as median (Q1, Q3), where Q1 and Q3 are the first and third quartiles, respectively. This format is used because of the non-normal distribution. *n* indicates the number of patients.				

The present letter examined serum IL-6 levels in children and adolescents with asthma and their associations with BMI and the presence of obstructive disorders for the first time. The significantly higher serum IL-6 levels in the overweight and obese BA patients compared with underweight and normal weight BA patients likely reflect systemic low-grade inflammation from excess adipose tissue. This finding is supported by a statistically significant direct correlation between serum IL-6 levels and zBMI. Previous studies by White *et al*^[8]^ and Maffeis *et al*^[9]^ also reported increased IL-6 concentrations, which align with our results. Additionally, research by Han *et al*^[10]^ identified IL-6 and adiponectin as potential biological mediators linking obesity and asthma in children when exploring common genetic components and potential biomarkers of this relationship. These findings suggest that the observed relationship of IL-6 with BMI and bronchial patency in children and adolescents with asthma in combination with overweight and obesity, which we found in a clinical study, is likely not coincidental.

IL-6 levels were influenced by the presence or absence of obstructive disorders, which were identified using spirometry based on an FEV_1_/FVC Z-score less than –1.645. In patients with obstructive disorders, IL-6 levels were statistically significantly increased in children with overweight and obesity, compared with those with underweight and normal weight. In patients without obstructive disorders, no significant differences in IL-6 concentrations were found between children with low/normal body weight and those with overweight and obesity. This may indicate the potential role of non-type 2-dependent inflammatory mechanisms in the development of obstructive patterns in children with overweight and obesity.

However, the present study has several limitations. It was conducted at a single center, and patients were not randomly recruited. Furthermore, the cross-sectional design limits our ability to draw conclusions about causality. Including normal-weight and overweight children and adolescents without BA as controls would strengthen future studies.

Yours sincerely,Regina Niyazovna Khramova^1^, Tatyana Ivanovna Eliseeva^1,✉^, Dmitriy Yuryevich Ovsyannikov^2^, Maksim Aleksandrovich Karpenko^2^, Elena Valerjevna Tush^1^, Svetlana Viktorovna Krasilnikova^1^, Daniil Vitalievich Azarnov^2^, Sofya Alekseevna Teremova^2^, Nailya Iskhakovna Kubysheva^3^, Оlga Vladimirovna Khaletskaya^1^, Natalia Anatolievna Geppe^4^ ^1^Department of Hospital Pediatrics, Privolzhsky Research Medical University of the Ministry of Health of the Russian Federation, Nizhny Novgorod 603005, Russia;^2^Department of Pediatrics, Medical Institute, Peoples' Friendship University of Russia (RUDN University), Moscow 117198, Russia;^3^Neurocognitive Research Laboratory, Kazan Federal University, Kazan 420000, Russia;^4^Clinical Institute of Children's Health named after N.F. Filatov, Sechenov First Moscow State Medical University, Moscow 119992, Russia. ^✉^Corresponding author: Tatyana Ivanovna Eliseeva. E-mail: eliseevati@yandex.ru.
